# Case Report: A young female with co-morbid SLE and atypical hemolytic uremic syndrome carrying *IRF5* risk variants

**DOI:** 10.3389/fimmu.2026.1966450

**Published:** 2026-10-14

**Authors:** Huili Zhou, Wei Liu, Tingting Sun, Li Zhang, Weixia Sun, Wanning Wang

**Affiliations:** Nephrology Department of the First Hospital of Jilin University, Changchun, Jilin, China

**Keywords:** atypical hemolytic uremic syndrome, case report, eculizumab, *IRF5*, systemic lupus erythematosus

## Abstract

Atypical hemolytic uremic syndrome (aHUS) is a thrombotic microangiopathy and primarily caused by abnormalities in the regulation of the alternative complement pathway, with triggers including infection, pregnancy, surgery and autoimmune disorders like systemic lupus erythematosus (SLE). This paper presents a case of a 21-year-old female carrying a SLE susceptibility variant (c.-12 + 198G>T) in the *IRF5*, who presented with concurrent manifestations of SLE and aHUS following diarrhea. The uniqueness of this case lies in the fact that comprehensive whole-exome sequencing revealed no known pathogenic mutations in complement genes (such as *CFH, CFI, C3*, etc.), yet identified two heterozygous variants in *IRF5*, a core gene of the interferon pathway (the c.-12 + 198G>T risk variant and the c.179A>G missense variant). Following targeted therapy centered on eculizumab, the patient achieved hematologic remission, but the kidney disease progressed to end-stage renal disease (ESRD), and maintenance hemodialysis was initiated. This case suggests that the genetic susceptibility underlying aHUS occurring in the context of SLE may extend beyond the classical complement pathway, and that excessive activation of the interferon pathway may be a key factor in triggering a complement activation ‘storm’ that manifests as aHUS. This observation suggests a possible link between IRF5-related interferon signaling and complement-mediated TMA, and highlights the need for further functional studies. It also emphasizes the importance of early recognition of aHUS and initiation of complement-inhibiting therapy in cases of SLE complicated by TMA.

## Introduction

Atypical hemolytic uremic syndrome (aHUS) is a thrombotic microangiopathy (TMA) caused by abnormalities in the regulation of the alternative complement pathway; pathogenic variants in complement regulatory genes such as *CFH*, *CFB*, *CFI* and *C3* can be detected in approximately 50–60% of patients (1, 2). However, in cases of aHUS that occur in the context of autoimmune diseases, particularly systemic lupus erythematosus(SLE), the genetic basis often differs from that of classic hereditary aHUS: patients typically do not carry single-gene mutations in complement genes, but rather carry genetic variants associated with susceptibility to autoimmune diseases, which indirectly lead to complement system dysfunction through immune dysregulation when triggered by specific environmental factors (3, 4). *IRF5* (interferon regulatory factor 5) is one of the most important susceptibility genes for SLE; the intronic single nucleotide polymorphism (SNP) rs2004640 (c.-12 + 198G>T) has been shown by genome-wide association studies (GWAS) to be significantly associated with the risk of developing SLE, a mechanism involving the excessive activation of the type I interferon pathway (5). However, there are currently no published reports indicating whether *IRF5* risk variants are associated with the occurrence of aHUS in patients with SLE. Here we report a severe female patient with SLE complicated by aHUS, and genetic testing identified a risk variant in the *IRF5* gene. Taking the genetic test results as our starting point, we retrace the progression of her clinical symptoms, explore how a genetic predisposition to SLE may have provided a ‘fertile ground’ for the development of aHUS, and analyze the implications of this finding for clinical diagnosis and management.

## Case presentation

A 21-year-old unmarried female was admitted due to oliguria, generalized edema for more than two weeks after diarrhea. Two weeks prior to admission, the patient developed diarrhea (5–10 times per day) for 4 days after ingestion of unclean food, followed by oliguria (minimum urine volume < 400 mL per 24 hours), eyelid edema, and subsequent generalized pitting edema. She was found to have new-onset hypertension for 10 days, with the highest blood pressure up to 160/120 mmHg. Fundoscopic examination was not performed, which is a limitation. However, there was no clinical evidence of hypertensive encephalopathy or other end-organ damage. The rapid normalization of platelet count and LDH after eculizumab, independent of blood pressure control, supported complement-mediated TMA as the primary mechanism. Five days ago, she presented to our hospital and was found to have elevated serum creatinine. She was then referred to the Department of Rheumatology and Immunology, where she was diagnosed SLE and received high-dose intravenous glucocorticoid pulse therapy. However, her serum creatinine continued to rise with persistent oliguria. For further diagnosis and treatment, she was admitted to our department under the impression of “lupus nephritis”.

During the course of the disease, she denied photosensitivity but complained of cough (worse in the morning) without sputum, occasional dizziness, headache, blurred vision and poor appetite. Past medical history: The patient had a history of thrombocytopenia of unknown cause for more than 2 years. She was admitted to the Hematology Department and received oral glucocorticoids for 1.5 months and eltrombopag for 1.5 years. Her platelet counts gradually returned to normal after treatment. ADAMTS13 activity was not measured during that earlier episode. However, the absence of microangiopathic hemolytic anemia, renal impairment, or neurological symptoms at that time, together with the sustained response to glucocorticoids and eltrombopag, made immune thrombocytopenia (ITP) or SLE-related immune cytopenia more likely than TTP. Nevertheless, an early episode of TTP could not be definitively excluded. In the physical examination, scattered ecchymoses distended abdomen, and pitting pretibial edema were positive. In the laboratory examinations, serum creatinine and blood urea nitrogen elevation, hypoalbuminemia, bicytopenia (anemia and thrombocytopenia) were observed.

Family and psychosocial history: There was no family history of autoimmune disease, hereditary kidney disease, or TMA. The patient was a college student. No significant psychosocial stressors were reported. Detailed psychosocial assessment was not performed, All blood and urine analysis results are presented in **Table 1**.

**Table 1:** Biological and immunological analysis at admission for acute renal injury.

Complete blood count	Value	Normal range
WBC (10^9/L)	5.48	3.5-9.5
RBC (10^12L)	2.36	3.8-5.1
HGB (g/L)	71	115-150
PLT (10^9/L)	8	125-350
PTA (%)	125.4	80-120
Kidney function		
Urea (mmol/L)	11.91	2.6-7.5
Creatinine (umol/L)	310.8	41-73
Uric acid (umol/L)	703	155-357
eGFR (ml/min)	17.66	>90
Immunological analysis		
C3 (g/L)	0.3	0.7-1.4
C4 (g/L)	0.03	0.1-0.4
Anti-dsDNA (IIF)	1:10 positive	<1:10
ADAMTS 13	–	–
C5b-9 (ng/mL)	>1580	75-219
Others		
LDH (U/L)	897	120-250
Shiga toxin-producing Escherichia coli	–	–

*PTA,* Prothrombin Activity; *ADAMTS 13*, A disintegrin and metalloprotease with thrombospondin Type 1 motifs, 13 member; *LDH*, Lactate dehydrogenase.

Given that antiphospholipid syndrome (APS) and catastrophic antiphospholipid syndrome (CAPS) are important differential diagnoses in patients with SLE-associated thrombotic microangiopathy, antiphospholipid antibody testing was performed. Lupus anticoagulant, anticardiolipin antibodies (IgG/IgM), and anti-β2 glycoprotein I antibodies (IgG/IgM) were negative. No clinical evidence of thrombosis or pregnancy morbidity fulfilling APS classification criteria was identified.

Hematological pathology analysis: Blood smear classification: Increased proportion of segmented granulocytes, accounting for 84%; one erythroblast was observed per 100 nucleated cells; mature red blood cells varied in size; spherocytes accounted for 2%; fragmented red blood cells accounted for 3% (uneven distribution); platelets were scattered; a small cluster of platelets was visible at the end of the smear.

She underwent whole-exome sequencing (including the mitochondrial genome, totaling 22,223 genes). The interpretation of the test results is divided into three levels (**Table 2**): The risk variants explicitly associated with SLE are summarized in **Table 2**.

**Table 2:** Risk variants explicitly associated with SLE.

Gene	Chromosomal location	Variant information	SNP ID	Homozygous/heterozygous	Pathogenic classification	Clinical significance
*IRF5*	chr7:128578301	c.-12 + 198G>T(Intron region)	rs2004640	het	Risk variation	Susceptibility to SLE: OR = 1.47 (heterozygous), OR = 2.01 (homozygous)
*IRF5*	chr7:128582314	c.179A>G(p.Asp60Gly)	rs201487893	het	Uncertain (REVEL=0.906)	Bioinformatics predicts toxicity; pathogenicity to be determined

The *IRF5* c.-12 + 198G>T (rs2004640) variant is the most significant genetic finding in this case. This variant is located within intron 1 of the *IRF5*, creating a novel splice donor site that leads to the production of multiple *IRF5* transcripts and increased IRF5 protein expression, which has been associated with increased type I interferon pathway activity in SLE (5).

### Analysis results for classic aHUS complement pathway genes

To elucidate the genetic background of aHUS, we performed whole-exome sequencing on the patient and focused our analysis on known aHUS-associated genes, including *CFH*, *CD46 (MCP)*, *CFI*, *C3*, *CFB*, *THBD* and *DGKE*. The results showed that no pathogenic or likely pathogenic variants were identified in these classic complement pathway-related genes. This negative result strongly suggests that the patient’s aHUS is not caused by a traditional hereditary complement gene defect, but is more likely secondary to an immunodysregulatory state triggered by their active SLE.

Among the ‘other reportable variants’ identified in whole-exome sequencing, two heterozygous variants of uncertain significance (c.3575G>A and c.1930G>T) were detected in the *NPHP4*, and heterozygous variants of uncertain significance were also found in *ATP6V0A4*, *DZIP1L* and *GFRA1.* These genes are associated with renal tubular development or ciliopathies, and none have been linked to complement-mediated TMA or aHUS. The two IRF5 variants (rs2004640 and rs201487893) were the only variants identified in immune-regulatory genes. rs2004640 is a well-established SLE risk variant, whereas rs201487893 is a variant of uncertain significance with predicted toxicity but no functional data. Thus, no pathogenic variant in a known aHUS-causing gene was identified. The IRF5 risk variant remains the most plausible susceptibility factor for the autoimmune background, although it is not a direct aHUS-causing gene.

We hypothesize the following two-hit sequence, although it remains unproven:

Initial onset (genetic susceptibility phase): The patient carries the IRF5 rs2004640 risk variant, which may create a genetic context of increased type I interferon pathway responsiveness, potentially increasing susceptibility to autoimmune diseases. The thrombocytopenia that emerged ‘without any apparent cause’ two years ago may represent an initial manifestation of preclinical SLE.

Second trigger (environmental trigger phase): In the two weeks prior to admission, the patient experienced diarrhea and an active EBV infection … Viral infection, as a classic environmental trigger, activates the Toll-like receptor (TLR) signaling pathway. In the context of the IRF5 risk variant, this may further amplify the type I interferon response, potentially contributing to the progression of SLE from a ‘subclinical state’ to a ‘phase of fulminant activity’.

Treatment response and outcome: Following the initial intravenous methylprednisolone pulse therapy, the patient was transitioned to oral prednisone at an initial dose of 50 mg/day, which was tapered by 5 mg every two weeks based on clinical response. Hydroxychloroquine 0.2 g daily was continued for long-term SLE control. Eculizumab was administered weekly (**Figure 1**). The clinical course and treatment response are shown in **Figure 1**. After the first dose, platelet counts rose rapidly and LDH levels returned to normal within one week, plasma sC5b-9, which was >1580 ng/mL (normal range 75–219 ng/mL) at admission, normalized after two weeks of eculizumab therapy. These finding confirmed terminal complement blockade. However, renal function did not recover, and the patient progressed to end-stage renal disease (ESRD). Regular maintenance hemodialysis was initiated. No severe infectious complications occurred during concomitant immunosuppressive and eculizumab therapy. Routine long-term monitoring of sC5b-9 after discharge was not performed, which represents a limitation of this case.

**Figure 1:**
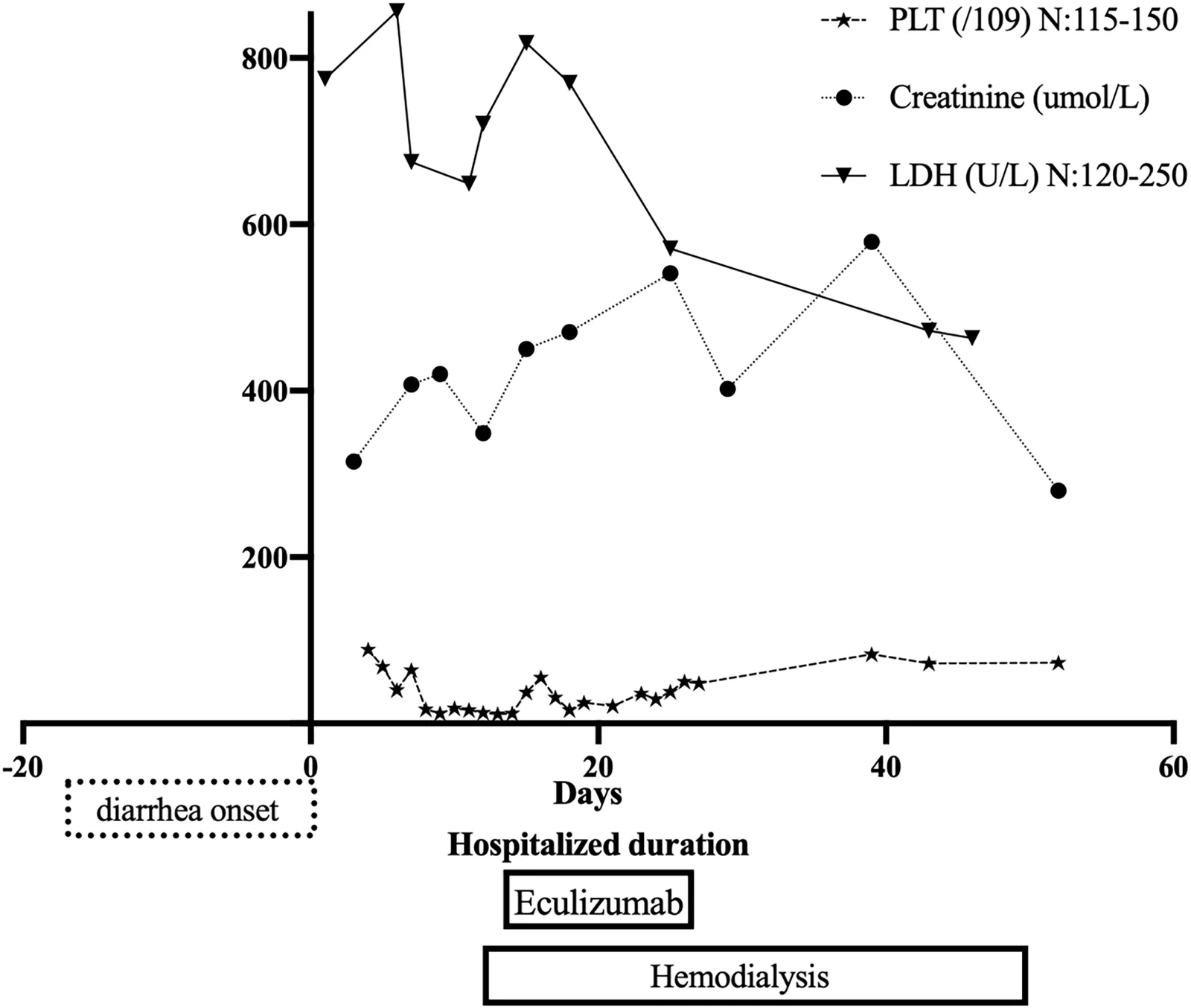
Treatment outcomes in the patient. Platelet count and LDH trends are shown from admission to follow-up. Key clinical events are annotated below the x-axis: diarrhea onset (Day -14), first hospital visit (Day -5), admission (Day 0), SLE diagnosis and pulse steroids (Day +3), eculizumab initiation (Day +7), hematologic remission (Day +14), ESRD (Week +6), and hemodialysis initiation (Week +8). Plasma sC5b-9 normalized after two weeks of eculizumab”.

## Discussion

Patients with SLE are prone to concurrent TMA due to immune abnormalities, complement activation, and organ damage (6). A Chinese study showed that among 148 patients with SLE, 36 were confirmed to have TMA by renal biopsy, most presenting with isolated renal TMA (29 cases), while only 2 cases developed TTP-HUS (4).

The principal novelty of this case is the identification of *IRF5* susceptibility variants in a patient with SLE-associated complement-mediated thrombotic microangiopathy (C-TMA) presenting with an aHUS phenotype, in the absence of complement gene mutations. Common *IRF5* risk haplotypes are well-established susceptibility factors for SLE and may alter *IRF5* splicing and expression, with some risk haplotypes being associated with increased circulating interferon-α activity (7–9). However, *IRF5* is not currently recognized as an aHUS-associated gene. Observations from a single patient cannot establish a genetic predisposition specific to the coexistence of SLE and aHUS. Therefore, we propose a hypothesis-not a proven mechanism-that *IRF5* susceptibility variants may have contributed to an interferon-high inflammatory background that increased vulnerability to complement-mediated endothelial injury, rather than directly causing aHUS.

We propose a hypothetical model in which the IRF5 risk variant may lead to increased IRF5 expression and type I interferon signaling, potentially creating an immune-inflammatory milieu in SLE and priming the alternative complement pathway (**Figure 2**). This proposed sequence is speculative and has not been functionally validated in this patient.

**Figure 2:**
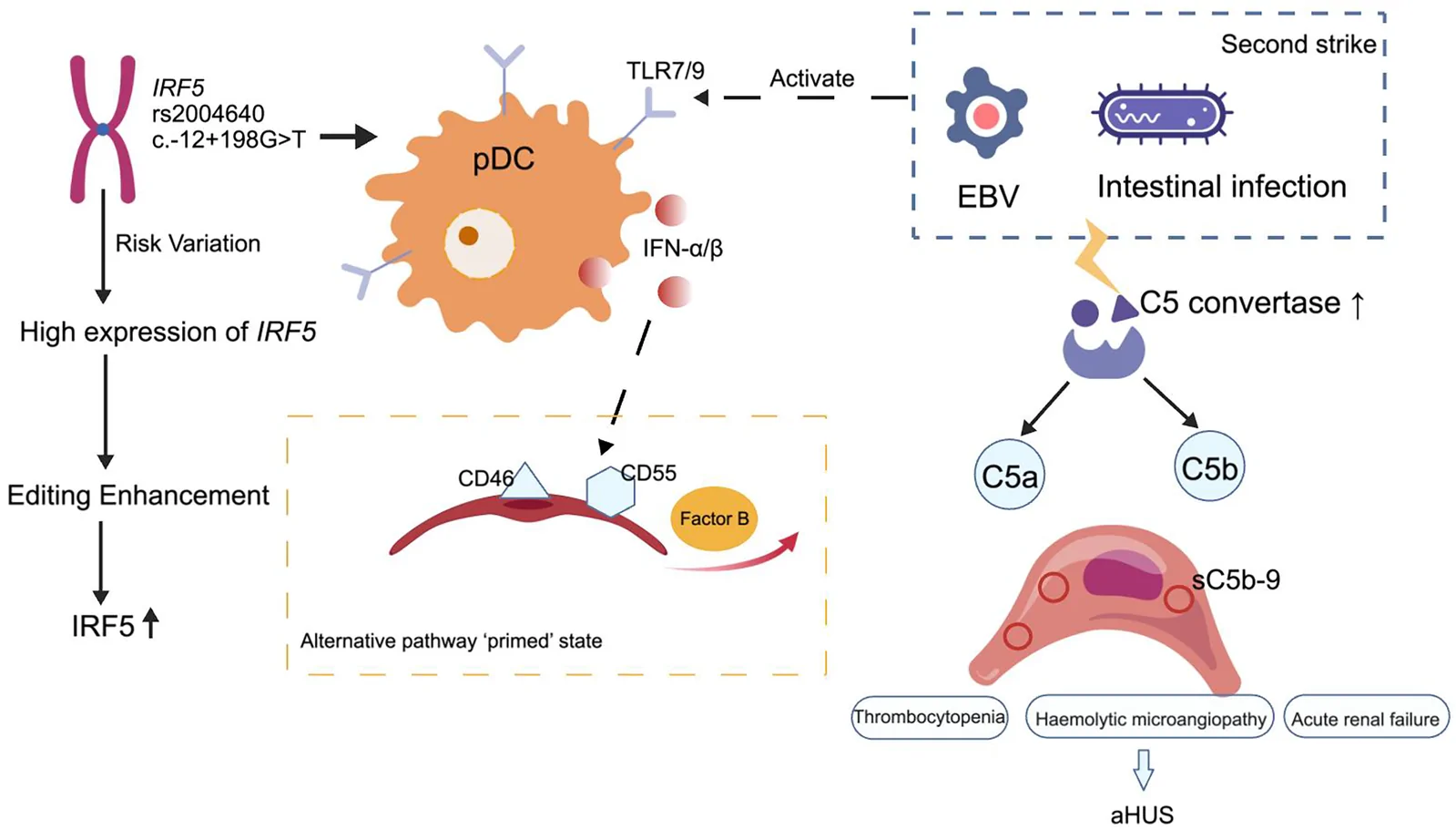
Hypothesized mechanistic model: *IRF5* risk variant may contribute to atypical hemolytic uremic syndrome (aHUS) in systemic lupus erythematosus (SLE) via type I interferon-mediated priming of the alternative complement pathway. This model is speculative and has not been functionally validated in this patient.

We fully acknowledge that there is currently no evidence supporting the use of type I interferon-targeted therapies (e.g., anifrolumab) in aHUS. In our patient, the acute TMA was driven by terminal complement activation, as evidenced by markedly elevated sC5b-9, which provided the rationale for eculizumab. The IRF5/IFN pathway, if involved, likely acts upstream as a disease modifier in SLE rather than as a direct therapeutic target for acute aHUS. Therefore, complement inhibition remains the appropriate treatment for the acute TMA episode, while SLE-directed immunosuppression addresses the underlying autoimmune background.

Given the absence of complement gene mutations, the patient’s aHUS is likely acquired and secondary to active SLE. In the absence of genetic ‘positive evidence’ of aHUS, plasma sC5b9 measurement serves as a critical bridge in diagnosis. In this case, significantly elevated sC5b9 levels (more than 6.5 times the upper limit of normal) directly confirmed terminal complement activation, thereby providing strong biological justification for the use of eculizumab.

The patient’s rapid response to eculizumab (platelet count recovery on the day following treatment and normalization of LDH levels within one week) further confirms that, even in the absence of complement gene mutations, C5 inhibitors remain effective provided that activation of the terminal complement pathway is confirmed. This is consistent with a systematic review of 30 patients with SLE-associated TMA, in which 93% experienced a favorable outcome, although the available evidence is derived predominantly from case reports and small uncontrolled series and is susceptible to selection and publication bias (10, 11). From a therapeutic perspective, adequate SLE control may reduce the risk of aHUS recurrence, potentially allowing earlier tapering or discontinuation of eculizumab (12–14). In our patient, SLE-directed maintenance therapy with oral prednisone, hydroxychloroquine, and regular hemodialysis was continued alongside weekly eculizumab. This combined approach was well tolerated, with no severe infectious complications, and may have contributed to the sustained hematologic remission.

## Conclusions

This case raises the hypothesis that IRF5 risk variants, through type I interferon pathway activation, may provide a molecular susceptibility background for the coexistence of SLE and aHUS. This hypothesis requires functional validation in future studies. The absence of pathogenic variants in classic complement genes suggests that the patient’s aHUS is a ‘secondary consequence’ of the immune dysregulation associated with SLE rather than a ‘primary complement gene defect’. This provides supportive evidence for the selection of treatment strategies (combining immunosuppression with complement inhibition) and the assessment of long-term prognosis (a lower risk of aHUS recurrence following control of SLE).

A key takeaway from this case is that, in patients with SLE complicated by TMA, a diagnosis of aHUS cannot be ruled out even if genetic testing for aHUS is negative. The detection of complement activation markers (sC5b-9) serves as a crucial link between clinical phenotype and treatment decisions. In the era of precision medicine, the value of genetic testing lies not only in ‘identifying pathogenic mutations’, but also in providing a systematic framework for the diagnosis and treatment of complex diseases through genotype-phenotype association analysis.

## The patient’s perspective

As a young patient, I felt extremely frightened and helpless as I went from suffering from diarrhea to being diagnosed with two serious conditions. Treatment with eculizumab led to a rapid improvement in my blood test results, which gave me a great deal of confidence. Although my kidneys did not ultimately recover and I require long-term dialysis, my doctor told me that the treatment had successfully managed the life-threatening acute phase. I have agreed to share my case in the hope that it may help others in a similar situation.

## Data Availability

The original contributions presented in the study are included in the article/**Supplementary Material**. Further inquiries can be directed to the corresponding author.

## References

[1] Azoulay E Knoebl P Garnacho-Montero J Rusinova K Galstian G Eggimann P . Expert statements on the standard of care in critically ill adult patients with atypical hemolytic uremic syndrome. Chest. (2017) 152:424–34. doi: 10.1016/j.chest.2017.03.055 28442312

[2] Yerigeri K Kadatane S Mongan K Boyer O Burke LLG Sethi SK . Atypical hemolytic-uremic syndrome: Genetic basis, clinical manifestations, and a multidisciplinary approach to management. J Multidiscip Healthc. (2023) 16:2233–49. doi: 10.2147/jmdh.s245620 37560408 PMC10408684

[3] Deng Y Tsao BP . Genetic susceptibility to systemic lupus erythematosus in the genomic era. Nat Rev Rheumatol. (2010) 6:683–92. doi: 10.1038/nrrheum.2010.176 21060334 PMC3135416

[4] Song D Wu LH Wang FM Yang XW Zhu D Chen M . The spectrum of renal thrombotic microangiopathy in lupus nephritis. Arthritis Res Ther. (2013) 15:R12. doi: 10.1186/ar4142 23320601 PMC3672792

[5] Sigurdsson S Nordmark G Göring HH Lindroos K Wiman AC Sturfelt G . Polymorphisms in the tyrosine kinase 2 and interferon regulatory factor 5 genes are associated with systemic lupus erythematosus. Am J Hum Genet. (2005) 76:528–37. doi: 10.1086/428480 15657875 PMC1196404

[6] Java A Kim AHJ . The role of complement in autoimmune disease-associated thrombotic microangiopathy and the potential for therapeutics. J Rheumatol. (2023) 50:730–40. doi: 10.3899/jrheum.220752 36642429

[7] Graham RR Kozyrev SV Baechler EC Reddy MV Plenge RM Bauer JW . A common haplotype of interferon regulatory factor 5 (IRF5) regulates splicing and expression and is associated with increased risk of systemic lupus erythematosus. Nat Genet. (2006) 38:550–5. doi: 10.1038/ng1782 16642019

[8] Niewold TB Kelly JA Flesch MH Espinoza LR Harley JB Crow MK . Association of the IRF5 risk haplotype with high serum interferon-alpha activity in systemic lupus erythematosus patients. Arthritis Rheum. (2008) 58:2481–7. doi: 10.1002/art.23613 18668568 PMC2621107

[9] Yu X Rehman AU Dang L Zhang X Liu J Xiong X . Interferon regulatory factor 5: a potential target for therapeutic intervention in inflammatory diseases. Front Immunol. (2025) 16:1535823. doi: 10.3389/fimmu.2025.1535823 40213550 PMC11983564

[10] Wright RD Bannerman F Beresford MW Oni L . A systematic review of the role of eculizumab in systemic lupus erythematosus-associated thrombotic microangiopathy. BMC Nephrol. (2020) 21:245. doi: 10.1186/s12882-020-01888-5 32605540 PMC7329551

[11] Legendre CM Licht C Muus P Greenbaum LA Babu S Bedrosian C . Terminal complement inhibitor eculizumab in atypical hemolytic-uremic syndrome. N Engl J Med. (2013) 368:2169–81. doi: 10.1056/nejmoa1208981 23738544

[12] Fakhouri F Fila M Provôt F Delmas Y Barbet C Châtelet V . Pathogenic variants in complement genes and risk of atypical hemolytic uremic syndrome relapse after eculizumab discontinuation. Clin J Am Soc Nephrol. (2017) 12:50–9. doi: 10.2215/CJN.06440616 PMC5220663 27799617

[13] Cataland SR Holers VM Geyer S Yang S Wu HM . Biomarkers of terminal complement activation confirm the diagnosis of aHUS and differentiate aHUS from TTP. Blood. (2014) 123:3733–8. doi: 10.1182/blood-2013-12-547067 24695849

[14] Chiu YY Nisihara RM Würzner R Kirschfink M de Messias-Reason IJ . SC5b-9 is the most sensitive marker in assessing disease activity in Brazilian SLE patients. J Investig Allergol Clin Immunol. (1998) 8:239–44. 9777539

